# The role and potential of umbilical cord blood in an era of new therapies: a review

**DOI:** 10.1186/s13287-015-0113-2

**Published:** 2015-07-02

**Authors:** Santiago Roura, Josep-Maria Pujal, Carolina Gálvez-Montón, Antoni Bayes-Genis

**Affiliations:** Heart Failure and Cardiac Regeneration (ICREC) Research Program, Germans Trias i Pujol Health Science Research Institute, Can Ruti Campus, Crta.Can Ruti-Camí Escoles s/n, 08916 Badalona, Spain; Cell Processing Laboratory, Edifici Giroemprèn, Pic de Peguera 11, Parc Científic i Tecnològic Universitat de Girona, 17003 Girona, Spain; Cardiology Service, Germans Trias i Pujol University Hospital, Crta.Can Ruti-Camí Escoles s/n, 08916 Badalona, Spain; Department of Medicine, Crta. Can Ruti-Camí Escoles s/n, Universitat Autònoma de Barcelona, 08916 Badalona, Spain

## Abstract

In light of pioneering findings in the 1980s and an estimation of more than 130 million global annual births, umbilical cord blood (UCB) is considered to be the most plentiful reservoir of cells and to have regenerative potential for many clinical applications. Although UCB is used mainly against blood disorders, the spectrum of diseases for which it provides effective therapy has been expanded to include non-hematopoietic conditions; UCB has also been used as source for regenerative cell therapy and immune modulation. Thus, collection and banking of UCB-derived cells have become a popular option. However, there are questions regarding the cost versus the benefits of UCB banking, and it also raises complex ethical and legal issues. This review discusses many issues surrounding the conservation of UCB-derived cells and the great potential and current clinical applications of UCB in an era of new therapies. In particular, we describe the practical issues inherent in UCB collection, processing, and long-term storage as well as the different types of ‘stem’ or progenitor cells circulating in UCB and their uses in multiple clinical settings. Given these considerations, the trend toward UCB will continue to provide growing assistance to health care worldwide.

## Introduction

The perspective regarding therapies based on multipotent ‘stem’ or progenitor cells is rather encouraging because of the large amount of research that recognizes human tissues as plentiful reservoirs of cells with a high capacity to regenerate damaged tissues [1–4]. Collection and banking of umbilical cord blood (UCB)-derived cells have become a popular option worldwide. However, there are questions regarding the cost versus the benefits of UCB banking, and it also raises complex ethical and legal issues [5–7].

This review discusses many issues surrounding the conservation of UCB-derived cells. In the context of other potential regenerative cell sources, we review the great potential and current clinical applications of UCB in the era of cell therapy. Briefly, we describe the practical issues inherent in UCB collection, processing, and long-term storage; UCB banking categories and ethical aspects; the relative benefits and economic burden associated with a rather long and costly procedure that is necessary to isolate and store cells for 25 to 30 years; and the different types of ‘stem’ or progenitor cells circulating in UCB and their uses in multiple clinical settings.

## Umbilical cord blood collection, processing, and cryopreservation

**Fig. 1:**
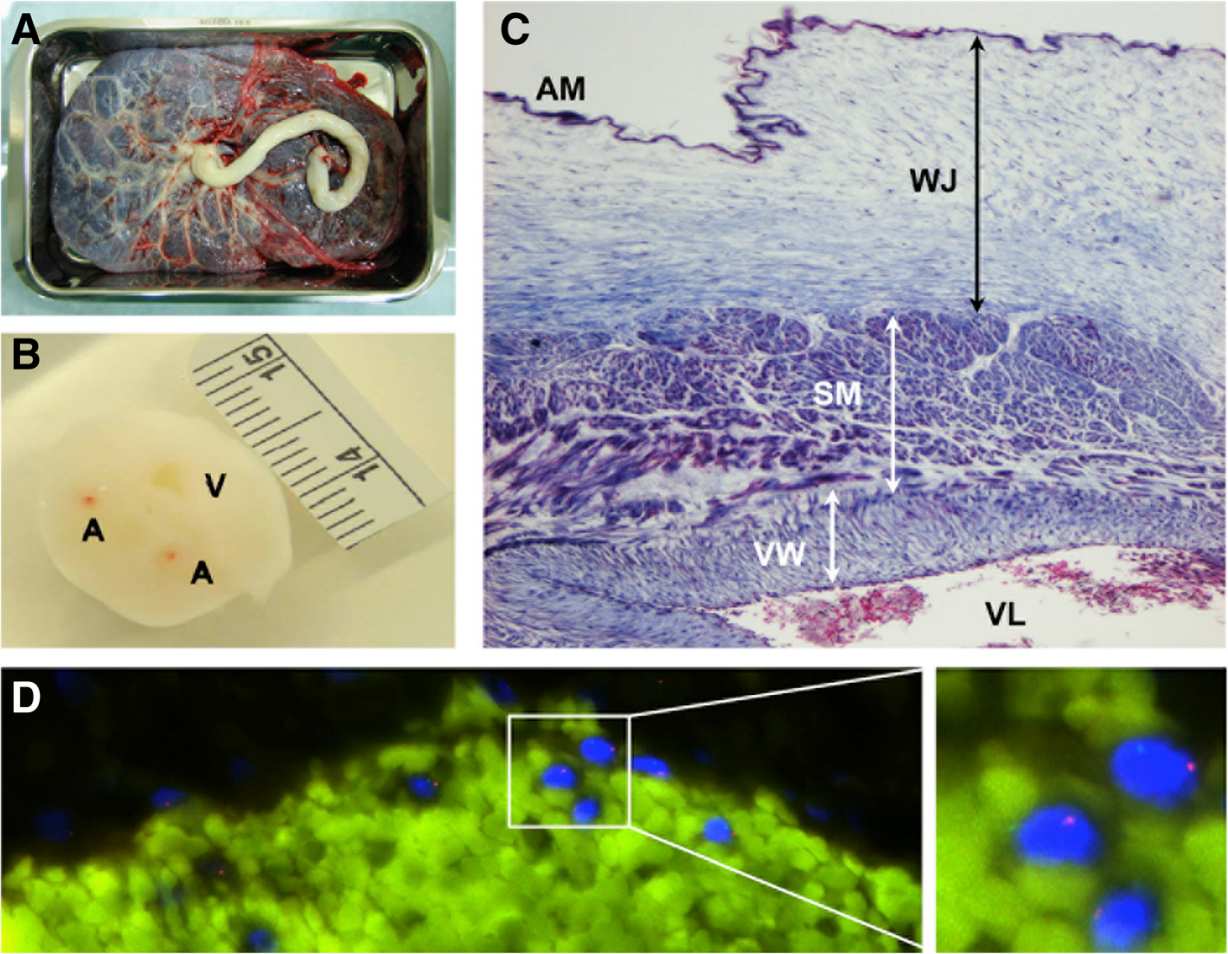
Umbilical cord: a tube containing highly ‘stem’ cell-enriched blood. Representative images show **a** the fetal face of a placenta from which an umbilical cord grows as a flexible, spongy-looking, tube-like structure usually around 55 cm or 2 feet, **b** a transversal section of umbilical cord showing two arteries (A) and one vein (V), and **c** a Masson’s trichrome staining of a complete umbilical cord microsection. At the structural level, amniotic membrane (AM), Wharton’s jelly (WJ), and smooth musculature (SM) associated with a blood vessel’s wall (VW) and lumen (VL) can be clearly distinguished. **d** The blood entrapped in the umbilical cord is recognized as a highly enriched source of valuable cells which can be visualized by, for example, fluorescence *in situ* hybridization using specific probes for X (green) and Y (red) chromosome

Because UCB is a highly enriched stem cell source (Fig. 1) [8], it is thought to be a helpful treatment for a number of genetic diseases, blood malignancies, and immune deficiencies. UCB may be also of medical use for a sick sibling or relative. Banking UCB is thus a way to preserve potentially life-saving cells that are usually discarded after the interruption of the blood supply from the umbilical cord to the newborn infant. Prior to collection, UCB donors are required to sign an informed consent form. At this time or alternatively up to 7 days before or 7 days after birth of the child, they are also tested for infectious diseases and microbial sterility. The precise timing for clamping and extracting the residual cord blood is important because umbilical vessels tend to collapse, according to Burton’s theory [9], as a consequence of (among other unknown mechanisms) the loss of blood flow (and thus pressure) and possibly temperature. The immediate consequence of the vascular occlusion is the coagulation of the trapped cord blood, which hinders the extraction of uncoagulated blood. Coagulation is one of the most cumbersome barriers to optimal sample extraction. The intent is to collect blood entrapped in the cord that would otherwise be released as a birth surplus. In addition, this procedure is non-invasive, not painful, and applicable to the vast majority of cases (vaginal or caesarean, induced or non-induced). Collection itself is a simple matter of venipuncture and drainage to a sterile container. Routinely, this procedure is completed within 5 minutes. However, UCB contamination predominantly occurs at this simple but critical point. During a vaginal birth, the external side of the cord (epithelial amniotic membrane) has been in close contact with vaginal or even colon-derived fluids, thus providing an entrance for contaminants throughout the venipuncture. UCB is not supposed to be contaminated, because it is an aseptic and closed system including only the baby, cord, and placenta; venipuncture is the only way to open this enclosed system.

Once the blood is collected, samples are included in a sterile bag (approximately 250 mL in size) that is then placed in an extraction kit in which temperature, pH, and CO_2_ and O_2_ levels, among other factors, depend only on time and external conditions. This encapsulated system, which must meet all regulatory shipping requirements, can regulate these basic features to a limited extent in order to maximize the number of cells that remain viable. Because cell survival is time-dependent, most collection facilities use external-induction-free boxes (with a temperature logging system) to isolate samples from the external influences of light and temperature. Transportation to cell processing laboratories is thus achieved within controlled and registered conditions.

**Fig. 2:**
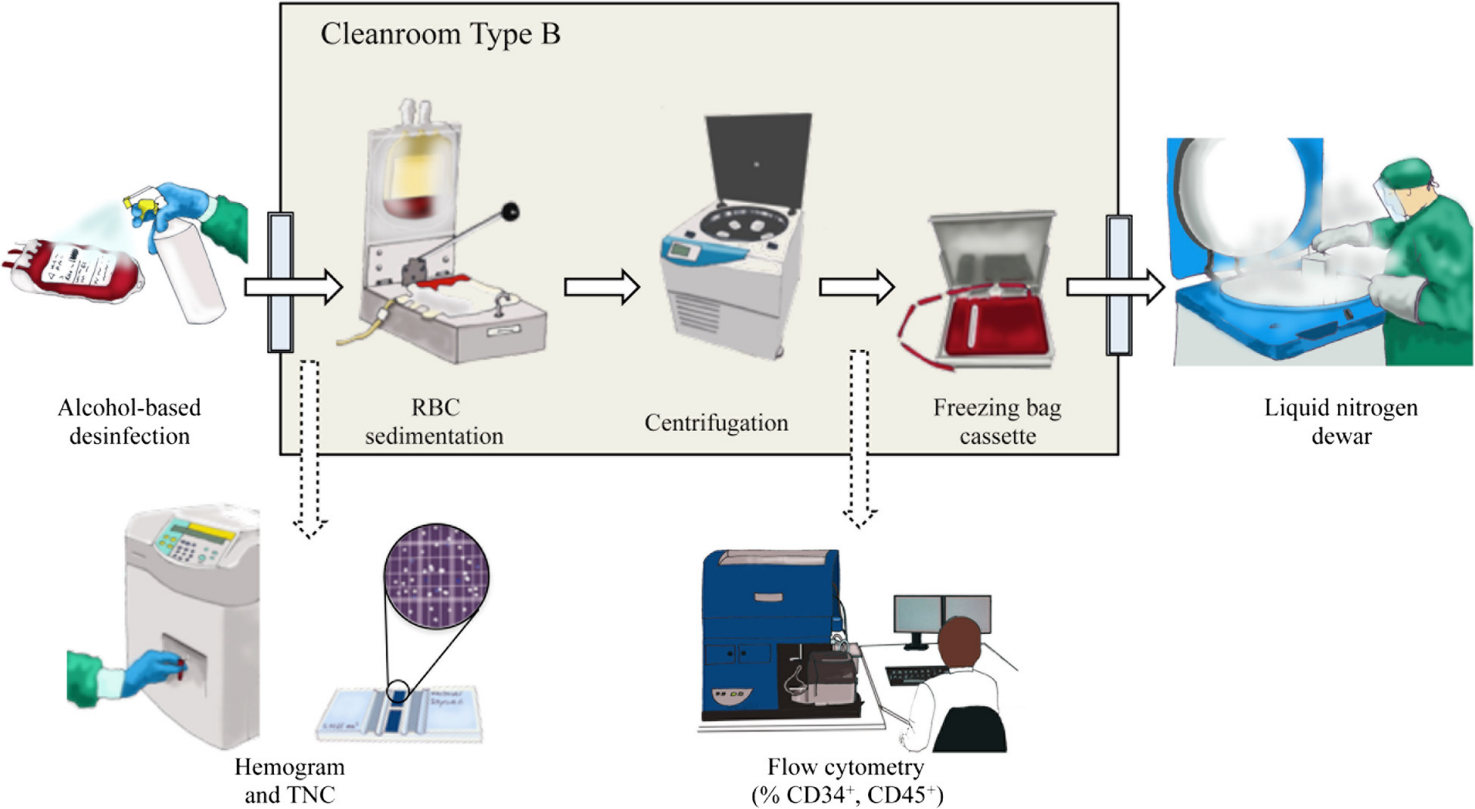
Umbilical cord blood collecting, processing, and banking: basic steps. In brief, once the umbilical cord blood extraction kit (temperature-insulated and padded for safety during transport) arrives at the processing laboratory, the blood bag’s external surfaces are disinfected prior to entrance in Cleanroom type B. Here, under highly sterile conditions, a pre-cryopreserved cell suspension enriched with mononuclear cells is collected following hydroxyethyl starch-based sedimentation of red blood cells (RBCs) and centrifugation. The resultant cell product is finally cryopreserved in a freezing bag cassette following a controlled-rate freezing process to slowly reduce the temperature to −180 °C and is stored in commercially available liquid nitrogen dewars. Routinely, quality controls based on the estimation of total nucleated cells (TNCs), percentage of CD34^+^ and CD45^+^ cells, and cell viability are performed throughout sample processing. The figure was designed and hand-drawn by CG-M

**Fig. 3:**
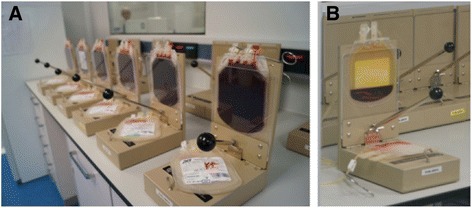
Sedimentation of red blood cells (RBCs). Representative photographs show umbilical cord blood collections at the beginning (**a**) and at the end (**b**) of the process of RBC sedimentation. Note that a yellow modified buffy coat, enriched in mononuclear cells, is obtained at the top of the bag. This procedure is central to reduce volume sample, storage space, and the cytotoxicity caused by the thawing of RBCs

Ideally, the separation and processing of large numbers of UCB units should be partially automated (Fig. 2). The majority of UCB collections are first red blood cell (RBC)-depleted prior to cryopreservation (Fig. 3). This step guarantees high rates of stem cell recovery because RBCs can make up more than half of the collection by volume, and only the mononuclear cell (MNC) fraction - where the stem cell population resides - is needed for banking. Furthermore, the volume reduction procedure, which is essential for cord blood banks to be economically efficient, rationalizes storage space and permits the reduction of dimethyl sulfoxide (DMSO) quantity in cellular products; it also diminishes the cytotoxicity caused by the thawing of RBCs [10–12]. Multiple methods have been used without significant loss of cell viability, including density gradient separation [13], sedimentation by gelatin [14], rouleaux formation induced by hydroxyethyl starch and centrifugation [15, 16], and differential centrifugation with expression of RBCs and plasma [17–19].

The methodology for UCB cryopreservation has been developed over time. Basically, UCB is processed and stored in either liquid- or vapor-phase nitrogen to maintain the viability and potential of the cell product [20]. The finding of Broxmeyer *et al*. [21] that controlled UCB cryopreservation for over 20 years had no significant effects on cell viability and function was proof-of-concept for the use of UCB banks in cell transplantation in humans. Others have also developed small-scale automated cryopreservation systems, such as the Mini-BioArchive system (Cesca Therpeutics Inc., Rancho Cordova, CA, USA), to provide cellular products adequate for UCB transplantation [22]. Methodologically, UCB banks use two methods to freeze cell products by using DMSO: red cell reduction (RCR) and plasma depletion (PD) [23]. Briefly, in the RCR method, cord blood is centrifuged in hetastarch or albumin to isolate 21 mL of cord blood containing mostly white blood cells; 4 mL of 50 % DMSO is added, and the resulting 25 mL of cell suspension is frozen. In the PD method, plasma is removed, and all of the cells are retained and frozen in 10 % DMSO. PD UCB units are cheaper to process but more expensive to store and somewhat more troublesome to thaw. However, when properly thawed and washed, PD UCB units have as many or more total nucleated cells (TNCs), CD34^+^ cells, and colony-forming units as well as higher cell engraftment rates; therefore, they may treat certain conditions, such as β-thalassemia, more effectively than RCR units [24]. As previously mentioned, DMSO is added immediately before the cord blood is frozen in order to protect cells by reducing the intracellular formation of ice crystals. However, DMSO concentrations of more than 1 % are toxic to blood cells for periods exceeding 30 minutes at 37 °C. For that reason, DMSO must be removed shortly after thawing to minimize adverse effects to transplanted patients [23].

The minimum acceptable pre-cryopreserved cell product is 2.5 × 10^7^ TNCs per kilogram of patient body weight [25]. Only approximately 10 % of banked units contain enough cells to be transplanted into an adult. In 2005, a team led by John Wagner published satisfactory results achieved in 23 recipients who received double partially human leukocyte antigen (HLA)-matched UCB units [26, 27]. Since then, this technique has been demonstrably efficient as a simple approach for overcoming cell dose limitations in older or heavier patients, in whom UCB transplantation with cell doses below this threshold is associated with slow hematopoietic recovery, poor engraftment, and high transplantation-related mortality [28, 29].

## Umbilical cord blood banking: options, ethics, and costs

A variety of UCB banks have been created worldwide in order to appropriately preserve donated units [6]. Initially, blood services were run by hospitals or non-profit institutions, which processed UCB samples and provided cells when needed. Accredited ‘public’ UCB banks were subsequently linked to national registries, which in turn were linked to international inventories. This coordination has favored the identification of the most suitable sample for each patient who requires a transplant [6]. Currently, since private companies have been offering UCB storage for their own or for family-related use, UCB banks are classified into the following categories: private or public and for-profit or non-profit. In general, public (non-profit) banks preserve cells derived from UCB and provide them altruistically; in contrast, private or commercial banks offer parents the service of exclusively preserving UCB stem cell products for expected progeny. Nevertheless, with many more UCB units banked privately than publicly in countries such as the US, other options, including mixed or hybrid private-public banks, have recently emerged [6, 7]. Preliminary analysis concludes that this hybrid UCB model offers limited benefit to the general public and also provides few advantages and potential disadvantages to private clients [30]. Indeed, whether the private option is preferable to the public option in UCB banking and whether it is possible to find a combined program of UCB banking with the best of both options remain topics of controversy. Nevertheless, as physicians, researchers, or even informed parents, we should make it our objective to inform new parents of all the options that are available since that is the most ethical thing to do. We must keep in mind that no other person should be allowed to decide the fate of your blood and other tissues (which belong to your child) and that public banking, private banking, or research use are equally valuable as a starting point.

Of course, concerning legal and regulatory aspects, many questions and ‘perils’ surrounding collection and storage of UCB can be identified and largely discussed. In brief, they include the following: informed consent, ownership, medical indications, claims related to medical benefits, allogeneic versus autologous use, legal frameworks, public versus commercial banks, financing systems, access and organization, quality assurance, traceability, relative costs, advertising, commercialization and patenting, personal data protection, privacy and confidentiality, and relationships between recipients, patients, doctors, and UCB banks [31]. For instance, a growing debate on donation versus self-preservation of cord blood has emerged [32]. As a result, a variety of national and international documents addressing these concerns have been drafted by national governments, parliaments, and authorities; bioethics committees; and national and international agencies, organizations, and societies. UCB donation and preservation are, however, endorsed by the major world religions [33].

But how much do UCB processing and banking cost? Even public cord blood banks state that the total cost of collecting, testing, processing, cryopreserving, and administering UCB-derived cells is considerable (up to $1,500 to $2,500 per UCB unit). Private banks usually charge a first-year processing fee ranging from about $1,400 to $2,300, plus annual storage costs of about $115 to $150. Private firms also proffer payment plans that range from no-interest installments paid over a few months to longer-term financing with interest. Special discounts if you prepay for longer periods of storage (5, 10, or 20 years) may be offered. Interestingly, a new trend in the industry is to offer a single all-inclusive price for 20 years of cell storage. In the long run, this is less expensive than the traditional price model with annual fees.

## Umbilical cord blood: a highly appropriate cell source for regenerative purposes

Given the current estimation of more than 130 million annual births, UCB is considered the most plentiful reservoir of regenerative cells for a large number of clinical applications [34]. In contrast to other unrelated donor cell sources, UCB is collected safely and painlessly, withstands long-term cryopreservation without loss of basic characteristics such as viability and function, and carries a low risk of transmitting viral infections and somatic mutations that could complicate patients’ clinical course after transplantation [35]. Furthermore, UCB can be used for allogeneic transplantation [36]. It is estimated that, as recently as 2012, about 115,000 solid organ transplants were performed worldwide [37]. Thus, alternative strategies to minimize maintenance immunosuppression in organ transplant recipients, including the use of regulatory cells [38] or induced tolerance by mixed chimerism [39], are evaluated. In terms of immunogenicity, some UCB-derived cell populations show inherent ‘immunoprivileged’ properties because they exhibit class I HLA antigens, and class II HLA antigens are seen only in response to interferon-gamma [40–42]. This lower UCB immunogenicity may be attributed to its immaturity, in contrast to adult stem cell sources. For these reasons, certain UCB-derived cell lineages are considered very useful tools for current regenerative medicine.

In terms of disadvantages, cell dosage and the possibility of delayed engraftment represent the main challenges to be fully resolved for the widespread use of UCB. For example, conventional UCB-based therapies are restricted when larger recipients are treated and for patients with diseases known to be resistant to engraftment because of a low number of hematopoietic cells per UCB unit. Thus, there is a need to develop more efficient *ex vivo* expansion strategies to increase the number of hematopoietic progenitor cells (HPCs) available from a single UCB unit for transplantation. For that reason, several culture conditions and automated devices have been developed in order to make UCB-derived cell products available to more patients, enhance the homing of transplanted cells, and allow more rapid post-transplant immune reconstitution [43–46]. Other developments consistent with good manufacturing practices for clinical application are expected, including serum-free media and a variety of reagents and potency assays to assess cell product activity. Nevertheless, because *ex vivo* culture can lead to spontaneous cell transformation and uncontrolled proliferative activity [47], an accurate preclinical evaluation of the safety profile of expanded cells is mandatory.

Many reports have indicated that a variety of cells with both *in vitro* and *in vivo* multilineage differentiation potential are contained in circulating UCB [48]. In general, these ‘stem’ or progenitor cells belong to hematopoietic or non-hematopoietic lineages and show higher *in vitro* proliferative ability than those from additional body sources such as bone marrow and adipose tissue [49, 50]. In particular, UCB-derived CD34^+^ cells transplanted *in vivo* exhibit greater repopulating ability than those extracted from bone marrow or mobilized peripheral blood. Kim *et al*. [51] compared the hematopoietic activities of CD34^+^ and CD34^−^ cells derived from human bone marrow and UCB and demonstrated that UCB is a better source of immature hematopoietic cells and that cells in its CD34^−^ fraction facilitate hematopoietic cell repopulation. This powerful hematopoietic capacity of UCB is attributed to its immaturity, in contrast to alternative adult cell sources.

As mentioned above, together with HPCs, UCB also contains non-hematopoietic cell types that can be readily isolated *ex vivo* by using established methods. Basically, these cell populations include mesenchymal stem cells (MSCs) and endothelial-like vascular progenitors, also termed endothelial progenitor cells (EPCs). In particular, MSCs comprise a population of multipotent progenitor cells (there is an estimated frequency of only 1,000 to 5,000 MSCs in a typical UCB unit of approximately 100 mL [52]), are capable of supporting hematopoiesis in bone marrow niches and differentiating into mesenchymal cell lineages (that is, osteogenic, adipogenic, and chondrogenic), and have immune modulatory properties. Several populations of mesenchymal-like stem cells with similar adhesion properties and antigen surface expression patterns but different pluripotency potential have been isolated from UCB. Originally, unrestricted somatic stem cells able to reprogram into mesodermal, endodermal, and ectodermal fates were isolated by Kögler *et al*. [53]. MSCs with more restricted pluripotency potential were subsequently characterized by others [54, 55]. In recent times, UCB-derived MSCs have garnered a great deal of attention for therapeutic purposes [56–60] and to preclinically predict the immunogenicity of prospective regenerative cells [61]. EPCs and additional cell types also labeled as EPCs (that is, outgrowth cells and circulating angiogenic cells) contribute to vascular development or reconstitution (or both) to varying degrees [62]. Other cells in UCB with regenerative potential are those expressing high levels of aldehyde dehydrogenase [63] and very small embryonic-like stem cells [64], even though the existence and characterization of the latter are still controversial [65].

## Multiple clinical settings for umbilical cord blood-derived cell therapy

Since the pioneering findings by Leary *et al*., Broxmeyer *et al*., and Gluckman *et al*. in the 1980s, there has been an increasing consensus that UCB can be used in clinical settings for hematopoietic cell transplantation [66]. As mentioned previously, several of the advantages of UCB for traditional transplantation (and some emerging) approaches are attributable to its collection at birth and the resultant immunological naivety. Broxmeyer *et al*. [8] were the first to firmly demonstrate that UCB is a rich source of transplantable HPCs. In the same year, Gluckman *et al*. [67] documented the first hematopoietic cell transplant to use UCB instead of bone marrow as the source of HPCs. Remarkably, the authors were able to reconstitute the hematopoietic system of a child with Fanconi anemia by using UCB from an HLA-identical sibling. However, the earliest evidence of the presence of relatively mature HPCs dates from 1974, when Knudtzon [68] observed *in vitro* growth of granulocytic colonies from circulating cells in human cord blood. About 10 years later, Nakahata and Ogawa [69] reported the presence of more primitive subpopulations of HPCs in UCB.

Evidence has continued to accumulate, and the establishment of a global network of UCB banks has contributed actively to allogeneic transplantation of HPCs in adults and children with hematological disorders. Recent estimates are that 600,000 samples are collected and more than 20,000 UCB units are distributed worldwide [34]. The success in HPC transplantation depends on the availability of a suitable donor. The best donor is a full HLA-matched sibling or unrelated donor. Unfortunately, on the basis of average family size, less than 30 % of patients will have a matched sibling donor [70]. Thus, the success rate of HPC transplantation is limited in part by immunological complications such as graft-versus-host disease (GVHD), graft rejection, and delayed immune reconstitution. Of course, immunological complications should not be a factor in the case of an autologous transplantation. For instance, acute GVHD is one of the major causes of morbidity and mortality after allogeneic cell transplantation. Risk factors for the development of acute GVHD include recipient age, cytomegalovirus serostatus, donor cell source, and HLA disparity. In this context, although the limited number of HPCs in a single cord-blood unit prevents its use in recipients with a larger body mass and induces delayed hematopoietic recovery and higher mortality, two partially HLA-matched units or double UCB grafts are increasingly chosen as alternatives to meet the minimum cell-dose requirement, mainly for those without an HLA-matched donor [71]. Indeed, despite one or two antigen disparities between donor and host, GVHD occurs with lower frequency after UCB transplantation compared with that observed after HLA-matched bone marrow or mobilized peripheral blood from unrelated donors because of the tolerogenic nature of UCB-derived T cells, MNCs, and especially immune regulatory cells [72, 73].

**Fig. 4:**
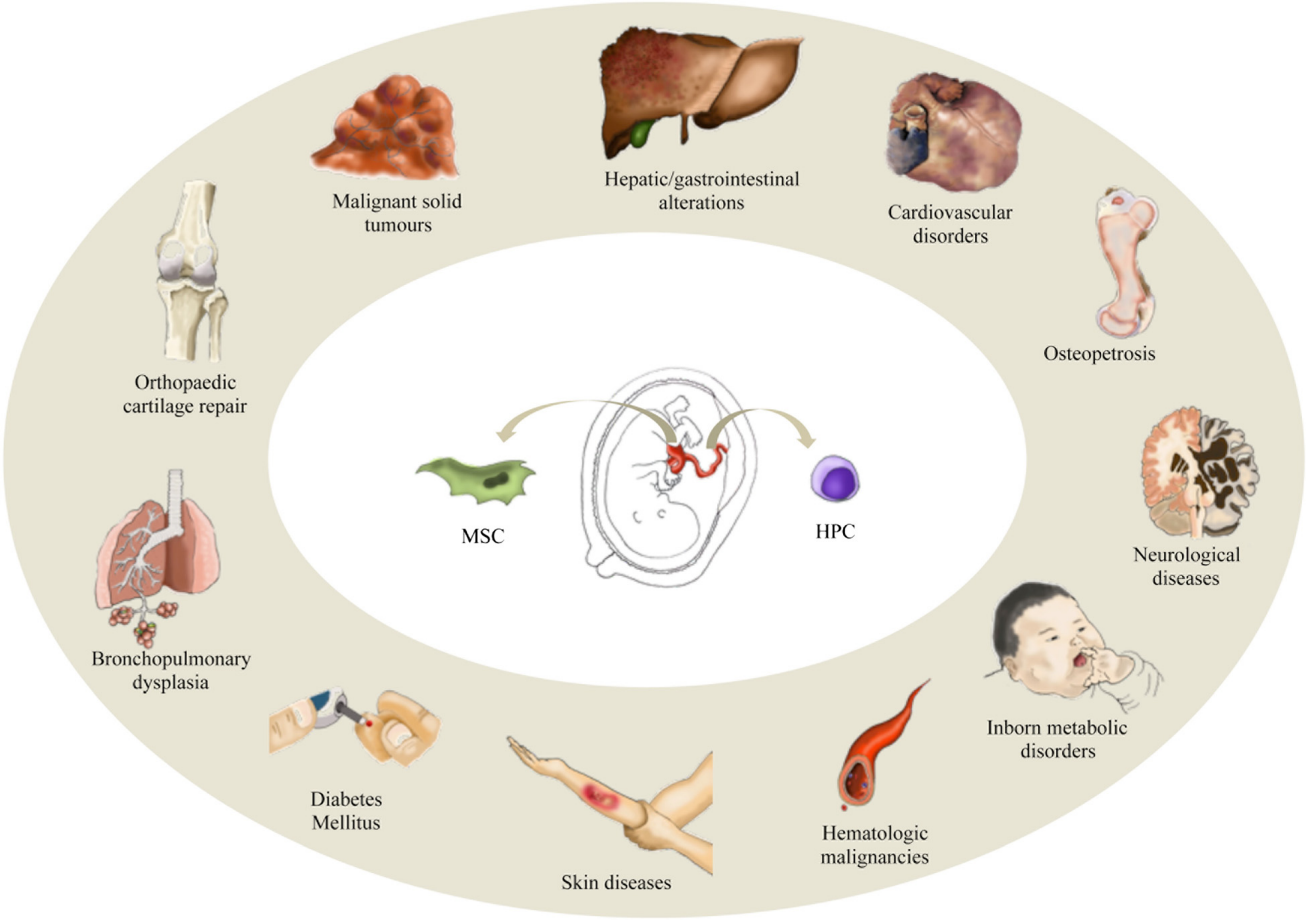
Current clinical applications of umbilical cord blood. The blood in the umbilical cord after the birth of a child is a readily available source of regenerative ‘stem’ or progenitor cells—for example, hematopoietic progenitor cells (HPCs) and mesenchymal stem cells (MSCs)—for use against many human diseases. The figure was designed and hand-drawn by CG-M

Although UCB is used mainly for HPC transplantation to treat blood disorders, the spectrum of diseases for which it is effective has been expanded to non-hematopoietic conditions, and UCB is also employed as a form of regenerative cell therapy or immune modulation [74, 75]. Table 1 and Fig. 4 summarize and illustrate the hematological and non-hematological diseases currently treated with UCB-derived cell products. Only a few of these clinical trials have already been completed and yielded results, whereas some have been stopped for some reason. For instance, the feasibility of the collection, preparation, and infusion of fresh autologous UCB cells for use in infants with hypoxic-ischemic encephalopathy was recently reported (NCT00593242) [76]. Allogeneic UCB therapy combined with recombinant human erythropoietin has demonstrated potential therapeutic efficacy for children with cerebral palsy (NCT01193660) [77]. Owing to UCB enrichment in vascular progenitors [78], angiogenesis has been induced in a 27-year-old woman with Behçet’s multisystemic disease [79] and in autistic children [80] who had received cells derived from this cell source. Lv *et al*. [81] have also reported preliminary results from a non-randomized, open-label, single-center phase I/II trial investigating the safety and efficacy of combined transplantation of human cord blood-derived MNCs and MSCs from umbilical cord in children with autism. These authors have concluded that the combination of the two cell types shows larger therapeutic effects than the transplantation of MNCs alone (NCT01343511). Regarding cerebral adrenoleukodystrophy, Miller *et al*. [82] have reported that progression of neurologic dysfunction of allogeneic UCB-derived hematopoietic cells post-transplantation depended significantly on the pre-transplantation Loes score and clinical neurologic status (NCT00176904, NCT00668564, and NCT00383448). In addition, Zhao *et al*. [83] have reported benefits in the context of diabetes mellitus type 2 with no safety and ethical concerns associated with conventional stem cell-based approaches. In particular, in that study, patients received one treatment with the Stem Cell Educator therapy in which a patient’s blood is circulated through a closed-loop system that separates MNCs from the whole blood, briefly co-cultures them with adherent cord blood-derived multipotent stem cells, and returns the educated autologous cells to the patient’s circulation (NCT01415726). However, autologous UCB infusion in children with diabetes mellitus type 1 has been safe and has induced changes in regulatory T-cell frequency but fails to preserve C-peptide (NCT00305344) [84]. Intratracheal transplantation of allogeneic UCB-derived MSCs in infants with bronchopulmonary dysplasia has proven to be safe and feasible but warrants a larger, controlled phase II study, as reported by Chang *et al*. [85] (NCT01297205). Interestingly, de Lima *et al*. [86] have studied cell engraftment in adults with hematological malignancies who received transplants of two cord blood units, one of which contained UCB that was *ex vivo*-expanded by using allogeneic MSCs. The authors concluded that transplantation of UCB-derived cells expanded with MSCs appears to be safe and effective and significantly improves cell engraftment (NCT00498316).

**Table 1:** Summary of major clinical trials using umbilical cord blood-derived cell products registered on www.clinicaltrials.gov

Disease	Identifier	Status	Cell origin
Alzheimer disease	NCT01297218	Completed	Allogeneic
Autism	NCT01343511	Completed	Allogeneic
Bronchopulmonary dysplasia	NCT01297205	Completed	Allogeneic
Cerebral palsy	NCT01072370	Recruiting	Autologous
	NCT01147653	Active, not recruiting	Autologous
	NCT01193660	Completed	Allogeneic
	NCT01528436	Completed	Allogeneic
Critical limb ischemia	NCT00518934	Unknown	Allogeneic
Diabetes mellitus type 1	NCT00305344	Completed	Autologous
	NCT00873925	Completed	Autologous
	NCT00989547	Active, not recruiting	Autologous
Diabetes mellitus type 2	NCT01415726	Completed	Autologous
Global development delay	NCT01601158	Completed	Allogeneic
Hematological malignancies	NCT00343798	Completed	Allogeneic
	NCT01175785	Active, not recruiting	Allogeneic
	NCT00498316	Recruiting	Allogeneic
Hypoplastic left heart syndrome	NCT01445041	Recruiting	Autologous
Idiopathic dilated cardiomyopathy	NCT01739777	Recruiting	Allogeneic
Inborn metabolic disorders	NCT00950846	Recruiting	Allogeneic
	NCT00920972	Recruiting	Allogeneic
	NCT01238328	Unknown	Allogeneic
	NCT00668564	Terminated	Allogeneic
	NCT00383448	Recruiting	Allogeneic
	NCT00176917	Completed	Allogeneic
	NCT00176904	Completed	Allogeneic
Liver failure caused by the hepatitis B virus	NCT01724398	Recruiting	Allogeneic
Malignant solid tumors	NCT00436761	Unknown	Allogeneic
	NCT00112645	Completed	Allogeneic
Neonatal hypoxic-ischemic encephalopathy	NCT00593242	Recruiting	Autologous
	NCT01649648	Recruiting	Autologous
Orthopedic cartilage repair	NCT01041001	Completed	Allogeneic
Osteoporosis	NCT00775931	Recruiting	Allogeneic
	NCT00638820	Terminated	Allogeneic
	NCT01087398	Unknown	Allogeneic
Skin diseases	NCT01443689	Unknown	Allogeneic
	NCT01033552	Recruiting	Allogeneic
Spinal cord injury	NCT01046786	Completed	Allogeneic
	NCT01471613	Completed	Allogeneic
Stroke	NCT01438593	Unknown	Allogeneic
	NCT01673932	Recruiting	Allogeneic
	NCT01700166	Withdrawn	Autologous
Traumatic brain injury	NCT01251003	Withdrawn	Autologous
	NCT01451528	Withdrawn	Allogeneic
	NCT01649648	Recruiting	Autologous

## Conclusions

As ethnic diversity increases in developing countries, it is imperative to find alternative stem cell sources when an adult-matched unrelated donor cannot be identified. At present, there are three alternative options: a partially HLA-mismatched unrelated donor, a haploidentical related donor, and a UCB stem cell product. Since the first UCB transplant in 1988, UCB has increasingly been employed as an alternative source of hematopoietic cells for transplantation in the treatment of blood diseases [34, 87]. Thus, the number of UCB banks worldwide has grown. This undeniable fact is being reinforced because of both the reduced alloreactivity of UCB, which allows greater HLA mismatching between donor cells and recipients, and GVHD incidence [88–90]. However, despite the application of volume reduction systems and improvements in homogenous recovery indices, several research groups have indicated that extended transport and storage times negatively affect UCB viability [91–93]. Therefore, the implementation of more highly qualified protocols of cell isolation, processing, cryopreservation, and expansion should be mandatory to guarantee that the optimal cell dosage is successfully transplanted.

Though used mainly for transplantation of HPCs, UCB has been extended to the treatment of non-hematopoietic disorders and immune modulation. This increasing interest in UCB enforcement has been mirrored in an increasing number of ongoing registered clinical trials. Although overall survival results for UCB transplantation are comparable to the results associated with matched unrelated donors, UCB transplants result in slow engraftment, delayed immune reconstitution, and increased opportunistic infections. While this may be a consequence of the lower cell dose in UCB grafts, it also reflects the relative immaturity of cord blood. Restricted cell numbers and the lack of availability of donor lymphocyte infusions also prevent post-transplant cellular immunotherapy to enhance donor-derived immunity for treating infections, mixed chimerism, or disease relapse. In this context, optimized strategies for engraftment and immune reconstitution after UCB transplantation are currently under investigation [94].

In summary, after an accurate analysis of published, current, and potential activities, UCB is one of the most active areas in human regenerative medicine. Indeed, because embryo-destructive transplantation medicine continues to elicit unfavorable opinion or prejudices, the trend toward UCB will probably continue to grow with the objective of assisting health care around the world.

## Abbreviations

DMSO: dimethyl sulfoxide
EPC: endothelial progenitor cell
GVHD: graft-versus-host disease
HLA: human leukocyte antigen
HPC: hematopoietic progenitor cell
MNC: mononuclear cell
MSC: mesenchymal stem cell
PD: plasma depletion
RBC: red blood cell
RCR: red cell reduction
TNC: total nucleated cell
UCB: umbilical cord blood
